# Tolterodine extended release is well tolerated in older subjects

**DOI:** 10.1111/j.1742-1241.2009.02108.x

**Published:** 2009-08

**Authors:** T L Griebling, S R Kraus, H E Richter, D B Glasser, M Carlsson

**Affiliations:** 1 Department of Urology and The Landon Center on Aging, University of KansasKansas City, KS, USA; 2 Department of Urology, University of Texas Health Science Center at San AntonioSan Antonio, TX, USA; 3 Division of Women’s Pelvic Medicine and Reconstructive Surgery, UAB Continence Center, University of Alabama at BirminghamBirmingham, AL, USA; 4 Pfizer IncNew York, NY, USA

## Abstract

**Objectives:**

To investigate the tolerability of tolterodine extended release (ER) in older subjects with overactive bladder (OAB).

**Methods:**

This was a retrospective analysis of pooled data from five large, randomised, double-blind, placebo-controlled trials. Subjects with OAB symptoms, including urinary frequency and urgency (and nocturia in two studies) with or without urgency urinary incontinence, received qd treatment with tolterodine ER (4 mg) or placebo for 8–12 weeks. Data were stratified *post hoc* by age group: < 65 (*n* = 2531), 65–74 (*n* = 1059) and ≥ 75 years (*n* = 573). Tolerability was assessed by evaluating the occurrence of adverse events (AEs). AE occurrences from each study were mapped to the MedDRA coding dictionary of preferred terms.

**Results:**

Discontinuation rates were slightly higher among subjects ≥ 75 years of age vs. those < 65 years of age; however, this was observed in subjects treated with placebo as well as tolterodine ER. Overall, there were no significant differences in the occurrence of dry mouth, headache, constipation, nausea, urinary tract infection, blurred vision, dry eye, dizziness and micturition disorder in older (65–74 or ≥ 75 years) vs. younger (< 65 years) subjects treated with tolterodine ER relative to placebo (treatment × age; all p > 0.1). Dry mouth was the only AE consistently associated with tolterodine ER treatment (< 65 years, 17%; 65–74 years, 16%; ≥ 75 years, 15%). The occurrence of all other AEs was ≤ 5% in most age and treatment cohorts. Most AEs were mild or moderate in all age and treatment cohorts.

**Conclusion:**

The nature and frequency of AEs associated with tolterodine ER treatment were similar across age groups in subjects with OAB, suggesting that tolterodine ER was not associated with an increased risk of AEs in older vs. younger subjects and, thus, is a suitable first-line pharmacotherapy treatment for OAB in this population.

**What’s known**

- Overactive bladder (OAB) is a prevalent and distressing syndrome characterised by urinary urgency, with or without urgency urinary incontinence, usually with increased daytime frequency and nocturia.
- OAB is associated with reduced health-related quality of life (HRQL) and related comorbidities, which may be more prevalent in the older population.

**What’s new**

- This retrospective analysis of pooled data from five large placebo-controlled clinical trials of patients with OAB examined the tolerability profile of tolterodine extended release in subjects aged 65–74 and ≥ 75 years compared with those aged < 65 years.

## Introduction

Overactive bladder (OAB) is a prevalent and distressing syndrome characterised by urinary urgency, with or without urgency incontinence, usually with increased daytime frequency and nocturia (1,2). Estimates suggest that 16.5% of adults in the United States, including 16.0% of men and 16.9% of women, have OAB (3). Similarly, OAB affects 16.6% of adults in Europe, including 15.6% of men and 17.4% of women (4). It is well established that the prevalence of OAB increases with age, as does the likelihood of experiencing urgency urinary incontinence (UUI) (3,4). In Europe, the prevalence of OAB in men and women has been estimated to be 3.4% and 8.7% among those 40–44 years of age, 18.9% and 16.9% in those 60–64 years of age and 41.9% and 31.3% in subjects ≥ 75 years of age respectively (4). A US-based study yielded similar age-related increases in OAB prevalence rates (3). Thus, the number of individuals affected by OAB is expected to increase because the older population is growing in the United States (5) and worldwide (6).

Overactive bladder imposes a considerable economic burden on society (7–10) and is associated with reduced health-related quality of life (HRQL) (11,12), as well as several comorbidities, such as depression (8,13), urinary tract infections (UTIs) (7,8,10) and falls/fractures (8,10). Older individuals may be particularly vulnerable to these comorbidities; however, the available evidence suggests that most older people with urinary incontinence do not receive treatment (14,15), despite the widespread availability of effective therapies.

Antimuscarinic agents, such as tolterodine, are currently the mainstay pharmacotherapy for OAB. Tolterodine is believed to work by reducing involuntary detrusor contractions (16,17) and has been shown to improve OAB symptoms (18–23) and the HRQL of individuals with OAB (24,25). Several studies have demonstrated that tolterodine is effective for the treatment of OAB symptoms specifically in older subjects (23,26–28). Antimuscarinics are generally well tolerated but are associated with certain adverse events (AEs), including dry mouth, blurred vision, constipation and dyspepsia (29).

Older individuals have special issues related to the tolerability of pharmacotherapeutic agents, such as increased comorbidities, polypharmacy and higher vulnerability to AEs. With the ageing demographical profile of the United States, it is important to understand whether the older patient may be at increased risk for AEs associated with antimuscarinic therapy for this condition. To that end, we investigated the tolerability profile of tolterodine extended release (ER) in subjects aged 65–74 years and ≥ 75 years with OAB relative to those aged < 65 years using pooled data from five large placebo-controlled clinical trials.

## Methods

In previously published reports examining the efficacy and tolerability of tolterodine in older subjects, the most commonly reported AEs were dry mouth, headache and constipation (23,26,27). For this retrospective evaluation, data from five large, randomised, double-blind, placebo-controlled clinical trials were pooled because of similarity in study design, inclusion/exclusion criteria and the enrolment of a large number of subjects. All subjects were enrolled for the treatment of OAB and received either placebo or tolterodine ER 4 mg qd. Participants were stratified into three age cohorts to assess the occurrence of commonly reported AEs and discontinuation rates. Subjects aged ≥ 65 years were separated from those aged < 65 years based on the definition of an ‘older’ person set forth in the Beers criteria (30). Additionally, subjects aged ≥ 65 years were stratified into 65- to 74-year and ≥ 75-year cohorts to investigate tolterodine ER tolerability in different age groups among older subjects. Existing studies using a 65-year age threshold to examine the efficacy of OAB treatment in older subjects have been criticised based on the concern that this age is no longer representative of the older population, and it has been suggested that more data on drug tolerability in individuals aged ≥ 75 years are needed (30). Primary results from three of the five studies (Pfizer-sponsored studies 007, 027 and 037) have been published (19,22,31); the results of the other two studies are publicly available at http://www.clinicalstudyresults.org.

Urinary frequency (≥ 8 micturitions per day) and urgency and/or UUI (≥ 5 episodes per week) were key inclusion criteria in each trial. Trial durations ranged from 8 to 12 weeks, and each trial was conducted in full accordance with the Declaration of Helsinki and International Conference on Harmonisation guideline and principles of Good Clinical Practice. The study protocol and all amendments for each trial were approved by the appropriate Institutional Review Boards and/or Independent Ethics Committees at each centre. All subjects in each trial provided written informed consent before entering the study.

The specific AEs examined in the current analysis included dry mouth, headache, constipation, nausea, UTI, blurred vision, dry eye, dizziness and micturition disorder. The data reflect all reported AEs, including those deemed related and unrelated to the study drug. All AEs spontaneously reported by the patient, directly observed by a clinician, or ascertained through direct questioning of the patient were included. Specific measurements of cognitive function were not conducted in any of these five individual trials. A logistic regression model was used to determine whether the estimated treatment effect (i.e. the difference between tolterodine ER and placebo) differed among the three age groups. The alpha level was set at 0.10, and Wald chi-squares for the treatment × age interaction terms are reported for each AE.

## Results

The characteristics of the study designs and subjects for the five trials are shown in Tables 1 and 2 respectively.

**Table 1:** Trial characteristics

	Clinical trial number				
	Study 007 (*N* = 1012)	Study 027 (*N* = 854)	Study 037 (*N* = 850)	Study 041 (*N* = 848)	Study 047 (*N* = 596)
Inclusion criteria	Frequency* and UUI†	Frequency* urgency with or without UUI† and SUI	Frequency*, urgency and nocturia, with or without UUI†	Frequency*, urgency and nocturia, with or without UUI†	Frequency* and urgency with or without UUI†
Exclusion criteria	SUI Total urinary volume > 3 l/day Significant hepatic or renal disease UTI (symptomatic or recurrent) Interstitial cystitis, haematuria, or BOO Recent electrostimulation or bladder training therapy Indwelling catheter or intermittent self-catheterisation Pregnancy, lactation, or failure by women of childbearing age to use reliable contraception Use of CYP3A4 inhibitors Treatment within 2 weeks of study with OAB therapy or anticholinergic drugs	Total urinary volume > 3 l/day Significant hepatic or renal disease UTI (symptomatic or recurrent) Interstitial cystitis, haematuria, or BOO Pregnancy, lactation, or failure by women of childbearing age to use reliable contraception Use of CYP3A4 inhibitors Non-surgical treatment for incontinence within 4 weeks of study Incontinence treatment within 2 weeks of study	SUI PVR > 200 ml Q_max_ < 10 ml/s Total urinary volume > 3 l/day Significant hepatic or renal disease UTI (symptomatic or recurrent) Interstitial cystitis, haematuria Recent electrostimulation or bladder training therapy Indwelling catheter or intermittent self-catheterisation Pregnancy, lactation, or failure by women of childbearing age to use reliable contraception Use of CYP3A4 inhibitors Treatment within 2 weeks of study with OAB therapy or anticholinergic drugs	SUI PVR > 200 ml Q_max_ < 10 ml/s Total urinary volume > 3 l/day Significant hepatic or renal disease UTI (symptomatic or recurrent) Interstitial cystitis or haematuria Recent electrostimulation or bladder training therapy Indwelling catheter or intermittent self-catheterisation Pregnancy, lactation, or failure by women of childbearing age to use reliable contraception Use of CYP3A4 inhibitors Treatment within 2 weeks of study with OAB therapy or anticholinergic drugs	SUI Interstitial cystitis or BOO UTI during run-in period Recent electrostimulation or bladder training therapy Pregnancy, lactation, or failure by women of childbearing age to use reliable contraception Allergy to tolterodine Use of CYP3A4 inhibitors Treatment within 2 weeks of study with OAB therapy or anticholinergic drugs

BOO, bladder outlet obstruction; CYP, cytochrome P450; OAB, overactive bladder; PVR, postvoid residual volume; Q_max_, maximal flow volume; SUI, stress urinary incontinence; UTI, urinary tract infection; UUI, urgency urinary incontinence.*Defined as ≥ 8 micturitions per day. †Defined as ≥ 5 episodes per week.

### Discontinuation rates

There was a trend for subjects ≥ 75 years of age to discontinue from the trials at a higher rate compared with subjects < 65 years of age (Table 2). However, this trend was observed in both tolterodine ER and placebo arms; discontinuation rates among subjects receiving tolterodine ER vs. placebo were 9% vs. 11% in subjects aged < 65 years, 8% vs. 10% in subjects aged 65 to 74 years and 12% vs. 15% in subjects aged ≥ 75 years respectively. The rates of discontinuation because of AEs were similar for both tolterodine ER and placebo groups (≤ 5%) and in all age categories, and there was no evidence of an age-related increase in the rate of discontinuation caused by AEs in subjects receiving tolterodine ER vs. placebo (Table 2).

**Table 2:** Subject characteristics

	Placebo			TOL ER		
	< 65 years (*n* = 1171)	65–74 years (*n* = 477)	≥ 75 years (*n* = 288)	< 65 years (*n* = 1360)	65–74 years (*n* = 582)	≥ 75 years (*n* = 285)
**Discontinuations, *n* (%)**						
Total*	126 (11)	49 (10)	43 (15)	115 (9)	45 (8)	35 (12)
Owing to an AE*	37 (3)	22 (5)	16 (6)	33 (2)	14 (2)	14 (5)
Men, *n* (%)	265 (23)	168 (35)	118 (41)	259 (19)	180 (31)	93 (33)
Mean (SD) age, years	50.8 (9.9)	69.0 (2.8)	79.1 (4.0)	50.7 (10.1)	69.1 (2.8)	78.9 (3.4)
Range	19–64	65–74	75–93	18–64	65–74	75–90
**Race, *n* (%)**						
White	1034 (88)	452 (95)	278 (97)	1224 (90)	555 (95)	278 (98)
Black	73 (6)	15 (3)	6 (2)	71 (5)	13 (2)	4 (1)
Asian	17 (2)	4 (1)	1 (< 1)	18 (1)	4 (1)	2 (1)
Other	47 (4)	6 (1)	3 (1)	47 (3)†	10 (2)	1 (< 1)
Mean (SD) body mass index, kg/m^2^	29.1 (6.9)	29.0 (5.9)	26.6 (4.5)	29.1 (9.0)	28.9 (5.7)	27.7 (12.4)
Range	15.1–63.9	16.8–49.9	15.7–47.2	16.5–211.5	17.1–60.3	15.8–198.8

TOL ER, tolterodine extended release; AE, adverse event; *Discontinuations and AEs include those related to and those not related to the study drug; †Includes 1 subject who did not specify race.

### Dry mouth

**Figure 1:**
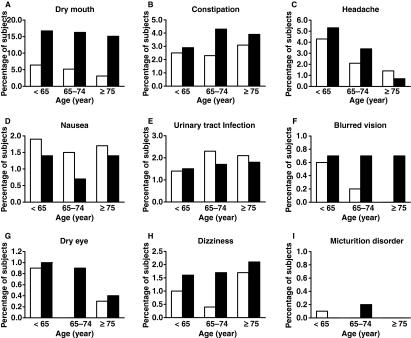
Occurrence of dry mouth (A), constipation (B), headache (C), nausea (D), urinary tract infection (E), blurred vision (F), dry eye (G), dizziness (H) and micturition disorder (I) by treatment and age group. TOL ER, tolterodine extended release

Dry mouth was the most commonly reported AE and the only AE consistently associated with tolterodine ER treatment (Figure 1A). Most instances of tolterodine ER-related dry mouth were of mild to moderate severity; the occurrence of severe dry mouth was only 1% in each age category. There were no treatment differences (χ^2^ = 2.5139; p = 0.285) in the occurrence of dry mouth among subjects who were aged < 65, 65–74 and ≥ 75 years (Figure 1A).

### Constipation

The occurrence of constipation was < 5% in all groups. Compared with placebo, there was a slightly higher rate of constipation in subjects receiving tolterodine ER treatment in each age cohort (Figure 1B). However, this treatment effect did not differ significantly among the < 65-year, 65- to 74-year and ≥ 75-year age cohorts (χ^2^ = 0.3162; p = 0.854).

### Headache

The occurrence of headache was generally low and tended to decrease with age regardless of treatment. Compared with the placebo group, headache was reported by slightly more subjects receiving tolterodine ER in the < 65-year and 65- to 74-year cohorts and by slightly fewer subjects in the ≥ 75-year cohort (Figure 1C). However, there were no age-related treatment differences (χ^2^ = 1.5548; p = 0.460) in the occurrence of headache.

### Nausea

The occurrence of nausea was low (< 2%) and appeared to be slightly higher in the placebo group and thus unrelated to tolterodine ER treatment. There were no treatment differences in the occurrence of nausea (χ^2^ = 0.1492; p = 0.928) among subjects aged < 65 years, 65–74 years and ≥ 75 years (Figure 1D).

### Urinary tract infection

Urinary tract infection did not appear to be related either to age or to tolterodine ER treatment (Figure 1E). Overall, the occurrence of UTI was < 3% in all age and treatment groups. There were no age-related treatment differences (χ^2^ = 0.7097; p = 0.701) in the rates of UTI.

### Blurred vision

The occurrence of blurred vision was ≤ 1% in all of the treatment and age cohorts; there were generally no differences in the occurrence of blurred vision between subjects receiving tolterodine ER and those receiving placebo (Figure 1F). Furthermore, there were no significant treatment differences (χ^2^ = 1.2175; p = 0.544) in the occurrence of blurred vision among subjects < 65 years, 65–74 years and ≥ 75 years.

### Dry eye

The occurrence of dry eye was < 1% in all treatment and age cohorts and appeared unrelated to tolterodine ER treatment (Figure 1G). There were no age-related treatment differences (χ^2^ = 1.4730; p = 0.479) in the rates of dry eye.

### Dizziness

The occurrence of dizziness did not exceed 2% in any treatment or age cohort (Figure 1H). There was no evidence that the occurrence of dizziness was associated with either age or tolterodine ER treatment, and there were no treatment differences in the occurrence of dizziness (χ^2^ = 1.8427; p = 0.398) among subjects aged < 65 years, 65–74 years and ≥ 75 years.

### Micturition disorder

Self-reported micturition disorder was experienced by < 1% of subjects regardless of age or treatment and was not associated with age or tolterodine ER treatment (Figure 1I). The difference between treatment groups in the rates of micturition disorder did not vary by age (χ^2^ = 1.1182; p = 0.572).

## Discussion

Although several studies have demonstrated that tolterodine ER is generally well tolerated in subjects with different demographical characteristics (23,26,27), none have investigated the tolerability of tolterodine ER in subjects aged ≥ 75 years relative to younger age groups. The current retrospective evaluation of pooled data from five studies provides further evidence that tolterodine ER is well tolerated in younger (aged < 65 years) and in older subjects (aged 65–74 or ≥ 75 years) with OAB. Importantly, the ratio of discontinuations because of AEs between tolterodine ER vs. placebo groups was similar among subjects aged < 65 years and subjects aged 65–74 or ≥ 75 years. However, it was found that AE-related discontinuation rates tended to be slightly higher in older subjects in both treatment groups. This finding is consistent with the notion that the older subjects may be more prone to tolerability and safety issues compared with their younger counterparts (32). Possible factors contributing to this finding may include changes in pharmacokinetics of antimuscarinics with age and polypharmacy in older patients (33). However, because the slight elevation in discontinuation rates was of similar magnitude among subjects ≥ 75 years in both placebo and tolterodine ER treatment groups, it is likely that this finding is primarily attributable to other factors, perhaps including comorbidities that lead to study discontinuation.

Not surprisingly, dry mouth, the most commonly reported AE associated with antimuscarinic agents (29), was consistently higher in subjects treated with tolterodine ER compared with placebo. Notably, neither the frequency nor the severity of dry mouth associated with tolterodine ER treatment was greater among subjects aged 65–74 or ≥ 75 years than among subjects aged < 65 years. Headache and constipation were also reported slightly more often by subjects receiving tolterodine ER compared with placebo. The occurrence of the other AEs investigated, including nausea, UTI, blurred vision, dry eye, dizziness and micturition disorder, was relatively low (< 3%), and none appeared to be associated with or attributed to tolterodine ER treatment. Moreover, none of these AEs was higher in older subjects, and no AEs occurred more frequently in subjects aged 65–74 or ≥ 75 years than among subjects aged < 65 years who were treated with tolterodine ER.

These data should be viewed within the context of their inherent limitations. For example, the analyses did not take into account the potential differences in medical comorbidities among the three age cohorts. Furthermore, because these studies were not designed for the purpose of comparing tolerability across age groups, outcomes for which older people may be particularly vulnerable were not assessed. The risk of cognitive impairment, from mild confusion to delirium and dementia, is of particular concern in older patients with a variety of pharmacological agents, including anticholinergic medications. Such concern is based on age- and health-related changes in neuropharmacological response and pharmacokinetics, as well as increased total anticholinergic burden related to greater likelihood of polypharmacy in the elderly (34,35). One published study found that anticholinergic use was associated with a greater risk for mild cognitive impairment (MCI) among elderly individuals (36); MCI was not evaluated in any of the five studies included in the current analysis. There is evidence that oxybutynin alters quantitative electroencephalogram (qEEG) activity in healthy volunteers (37) and may compromise cognitive activity in older patients (38,39). Tolterodine does not readily cross the blood–brain barrier (40) and exerts little impact on qEEG activity (37), although there have been isolated case reports of cognitive impairment (41–43). This issue deserves further investigation through large well-controlled clinical trials powered to assess this outcome.

The data presented in this report are consistent with the most recent Beers criteria (30), which provide guidance for appropriate prescribing practices in older adults. The Beers criteria do not list tolterodine as a medication to be avoided in older individuals except in those with clinically significant bladder outlet obstruction (BOO). This is based on a theoretic concern about increased risk of urinary retention (30). As stated in the prescribing information for tolterodine ER and other antimuscarinics (44–48), such agents must be used with caution in individuals of any age with clinically significant BOO. Thus, this appears to be a class effect rather than an age-related effect. Although not listed in the Beers criteria, antimuscarinic agents are also contraindicated in individuals with a history of unstable or untreated narrow-angle glaucoma (44–48), the prevalence of which is increased in older individuals.

These data are also consistent with previous studies in which tolterodine immediate release (IR) was shown to have a favourable tolerability profile in older subjects. In a double-blind placebo-controlled study with subjects aged ≥ 65 years, no differences were found in the occurrence of serious AEs between subjects taking placebo vs. tolterodine IR (1 or 2 mg bid) (26). Similarly, a logistic regression analysis of data from a large, open-label, observational study did not find any significant effect of age on the tolerability of tolterodine IR treatment (27). The results of a nursing home urinary incontinence management programme also suggest that tolterodine IR (1 or 2 mg bid) is well tolerated in this older population (28). The results of the current analysis suggest that tolterodine ER, which has better efficacy and tolerability than tolterodine IR (22), is also well tolerated in older individuals. This finding, combined with the observations that tolterodine ER is an effective and safe treatment for OAB, is important because the prevalence of OAB increases with age. Thus, the potential for differential susceptibility to these common AEs, as well as others, needs to continue to be explored (3,4).

## Conclusion

The results of the current analysis suggest that the tolerability profile of tolterodine ER is not diminished in older individuals (aged 65–74 years or ≥ 75 years) with OAB relative to younger individuals (aged < 65 years). Moreover, there was no increase in risk for commonly reported AEs in older vs. younger subjects. This suggests that tolterodine ER is suitable as a first-line pharmacotherapy treatment for OAB in older individuals.
